# Supplementary material for: Content-rich biological network constructed by mining PubMed abstracts
Source: BMC Bioinformatics. 2004 Oct 8;5:147. doi: 10.1186/1471-2105-5-147 (PMC528731; doi:10.1186/1471-2105-5-147)
Supplement: Additional File 2 — The original results of the above study (non-essential files are deleted to keep the file size under the limit set by BMC bioinformatics). [file 1471-2105-5-147-S2.bz2 › chilibotAdditionalFile2/dip20/214ID7790364E720/html/CD9_ITGB1.html]

 


 **CD9** and **ITGB1** 
  
Found 30 abstracts in PubMed, retrieved 20.  
 

 What does Google say? 
 PDF only 
| .edu only 

---

**Interactive relationship** (e.g. stimulation, inhibition, etc)

**Inhibitory relationship**- Anti CD29  [ **ITGB1** ]  MoAbs inhibited gel contraction, but anti  **CD9**  MoAbs counteracted this inhibitory effect of anti CD29  [ **ITGB1** ]  MoAbs.  Ref: 9812906 Arterioscler Thromb Vasc Biol, 1998
**Neutral relationship**- Immunoprecipitation of T cell lysates with anti  **CD9**  mAb followed by immunoblotting with mAb against various T cell molecules showed the association of  **CD9**  with CD3, CD4, CD5, CD2, CD29  [ **ITGB1** ]  and CD44.  Ref: 10590270 Int Immunol, 1999

**Non-interactive relationship** (e.g. studied together, co-existance, homology, etc.)

- Expressed proteins include  **CD9** , CD10, CD13, CD29  [ **ITGB1** ] , CD34, CD44, CD 49 d, CD 49 e, CD54, CD55, CD59, CD105, CD106, CD146, and CD166.  Ref: 11573204 J Cell Physiol, 2001
- Three patterns of antigen expression were detected 1 markers constantly positive in all cases analyzed  **CD9** , CD29  [ **ITGB1** ] , CD33, CD43, CD44, CD49d, CD49e, CD51, CD71, CD117, and Fc epsilon RI, 2 antigens that were constantly negative CD1a, CD2, CD3, CD5, CD6, CD11a, CD14, CD15, CD16, CD19, CD20, CD21, CD23, CD25, CD30, CD34, CD38, CD41a, CD42b, CD65, CD66b, HLA DR, and CD138, and 3 markers that were positive in a variable proportion of cases CD11b 50%, CD11c 77%, CD13 40%, CD18 20%, CD22 68%, CD35 27%, CD40 67%, CD54 88% and CD61 40%.  Ref: 9699944 Anal Cell Pathol, 1998
- It also contained signaling molecules such as Xyk Xenopus egg Src, Gq alpha, Ras, integrin beta 1  [ **ITGB1** ]  and  **CD9** .  Ref: 11861472 Development, 2002
- Data obtained are presented in concurrence with Clusters of Thymic Epithelial Staining CTES classification for various monoclonal antibodies recognizing CD antigens CD1, CD1a, CD6,  **CD9** , CD14, CD16, CD29  [ **ITGB1** ] , CD30, CD32, CD44, CD45RB, CD47, CD48, CD49a, CD49b, CD49c, CD49d, CD49e, CD49f, CD51, CD53, CD54, CD56, CD57, CD63, CD85, CD95, CD98, CD102, CD103, CD106, CD109, CD146, CD147, CD148, CD151, CD152, CD158a, CD158b, CD164, CD165, CD166 and for monoclonal antibodies 1B1 G7, A4, BD46, BLTZ, HP1C5, IND.64, M72, WU947 whose specifities are not yet defined.  Ref: 12725440 Ann Anat, 2003
- In the contractile phenotype, the levels of  **CD9** , CD81, another tetraspanin, and CD29  [ **ITGB1** ]  are approximately 50% of those found in the proliferative phenotype.  Ref: 9812906 Arterioscler Thromb Vasc Biol, 1998
- MLSC was able to measure antigens  **CD9** , CD29  [ **ITGB1** ] , CD36, CD41, CD42a, CD42b, and CD61 on platelets and determine an average of 2.3 x 10 5 7 x 10 4 platelets per microliter.  Ref: 11487384 Platelets, 2001
- Confirming our previous analyses on lung MC, foreskin MC reacted with MoAb against  **CD9** , CD29  [ **ITGB1** ] , CD33, CD43, CD44, CD45, CD46, CD51, CD54, CD55, CD58, CD59, CD61, and CD117.  Ref: 9764855 J Invest Dermatol, 1998
- Based on their immunophenotype, the cells are relatively homogeneous, with shared expression of integrin beta 1  [ **ITGB1** ] , the hyaluronate receptor, and the tetraspan protein  **CD9** , among other markers.  Ref: 12880371 Expert Opin Biol Ther, 2003
- Anti  **CD9** , CD26, CD29  [ **ITGB1** ] , CD31, CD36, CD44, CD49a, CD49b, CD49c, CD49d, CD49e, CD49f, CD54, CD55, CD56, CD62E &.  Ref: 10235320 J Pediatr Surg, 1999
- We studied the changes in expression of  **CD9**  and beta1 integrins CD29  [ **ITGB1** ] , VLA in human vascular smooth muscle cells VSMCs under in vitro culture conditions mimicking proliferative vascular diseases.  Ref: 9812906 Arterioscler Thromb Vasc Biol, 1998
- RESULTS In both groups of patients RA and OA, SyMC were found to react with MAb to IgE, SCF receptor c kit, CD117, as well as CD antigens likewise expressed in lung MC  **CD9** , CD29  [ **ITGB1** ] , CD33, CD43, CD44, CD45.  Ref: 9485081 Arthritis Rheum, 1998
- Double staining with toluidine blue and immunofluorescence using monoclonal antibodies mAbs revealed expression of stem cell factor SCF receptor c kit CD117,  **CD9** , CD29  [ **ITGB1** ] , CD33, CD43, CD44, CD54, and CD63 on HRMCs.  Ref: 9472805 Exp Hematol, 1998
- TNF alpha or IL 1beta did not change the expression of  **CD9** , CD13, CD16, CD23, CD29  [ **ITGB1** ] , CD31, CD32, CD35, CD45, CD61, or CD64 in BEAS 2B cells.  Ref: 9374108 Am J Respir Cell Mol Biol, 1997
- The proteins purified from GC by immunoaffinity chromatography using anti integrin alpha6 monoclonal antibodies were shown by Western blotting to include  **CD9**  as well as integrin beta1  [ **ITGB1** ] .  Ref: 10321801 Mol Hum Reprod, 1999
- PMP stimulation of ECs increased CD54 and CD63 but not  **CD9** , CD29  [ **ITGB1** ] , and CD31.  Ref: 11583705 Atherosclerosis, 2001
- Coimmunoprecipitation experiments showed physical association between  **CD9**  and CD29  [ **ITGB1** ] .  Ref: 9812906 Arterioscler Thromb Vasc Biol, 1998
